# Taxonomic Distribution of Cytochrome P450 Monooxygenases (CYPs) among the Budding Yeasts (Sub-Phylum *Saccharomycotina*)

**DOI:** 10.3390/microorganisms7080247

**Published:** 2019-08-08

**Authors:** Tomas Linder

**Affiliations:** Department of Molecular Sciences, Swedish University of Agricultural Sciences, 750 07 Uppsala, Sweden; tomas.linder@slu.se

**Keywords:** CYPome, enzyme, metabolism, orphan gene, yeast

## Abstract

Cytochrome P450 monooxygenases (CYPs) are ubiquitous throughout the tree of life and play diverse roles in metabolism including the synthesis of secondary metabolites as well as the degradation of recalcitrant organic substrates. The genomes of budding yeasts (phylum *Ascomycota*, sub-phylum *Saccharomycotina*) typically contain fewer families of CYPs than filamentous fungi. There are currently five CYP families among budding yeasts with known function while at least another six CYP families with unknown function (“orphan CYPs”) have been described. The current study surveyed the genomes of 372 species of budding yeasts for CYP-encoding genes in order to determine the taxonomic distribution of individual CYP families across the sub-phylum as well as to identify novel CYP families. Families CYP51 and CYP61 (represented by the ergosterol biosynthetic genes *ERG11* and *ERG5*, respectively) were essentially ubiquitous among the budding yeasts while families CYP52 (alkane/fatty acid hydroxylases), CYP56 (*N*-formyl-l-tyrosine oxidase) displayed several instances of gene loss at the genus or family level. Phylogenetic analysis suggested that the three orphan families CYP5217, CYP5223 and CYP5252 diverged from a common ancestor gene following the origin of the budding yeast sub-phylum. The genomic survey also identified eight CYP families that had not previously been reported in budding yeasts.

## 1. Introduction

The budding yeasts (phylum *Ascomycota*, sub-phylum *Saccharomycotina*) comprise over 1000 described species of predominantly unicellular fungi that include many species of industrial or medical importance [1]. At present, more than 350 species of budding yeasts have been subjected to genome sequencing. Although the gene content of a haploid budding yeast genome is fairly constant with average gene number of 6000–7000 [2], there exists significant diversity of gene inventory between yeast lineages at both the genus and family level [3]. While most genes of the common baker’s yeast *Saccharomyces cerevisiae* have established functions, the functions of the vast majority of genes that do not occur in *Sac. cerevisiae* remain to be determined. The assignment of biological functions to such “orphan genes” [4] remains the primary challenge of budding yeast genomics.

Cytochrome P450 monooxygenases (CYPs) are a large family of heme-thiolate enzymes (Pfam accession 00067) that are found throughout the tree of life [5]. Among the fungi, CYPs are involved in many metabolic processes including the synthesis of secondary metabolites as well as the degradation of complex organic substrates and xenobiotics. Budding yeast genomes contain relatively few CYP genes as a percentage of the total gene content compared to filamentous ascomycete and basidiomycete fungi [6]. Two possible reasons for this lower proportion of CYP-encoding genes is the limited number of metabolic pathways for secondary metabolite synthesis and complex substrate degradation within the budding yeast sub-phylum.

All CYPs are divided into ten distinct classes depending on the protein components involved in electron transfer to the CYP catalytic subunit [7]. Three classes of CYPs have been described in the fungi of which class II CYPs are the most common. Class II CYPs are membrane-bound proteins that are localized to the endoplasmatic reticulum and act in concert with an FMN/FAD-containing CYP reductase (CPR) and in some cases also a cytochrome *b*5 subunit [6]. CYPs are further classified into individual families based on amino acid sequence identity with the family inclusion threshold set at 40% sequence identity [8].

**Figure 1:**
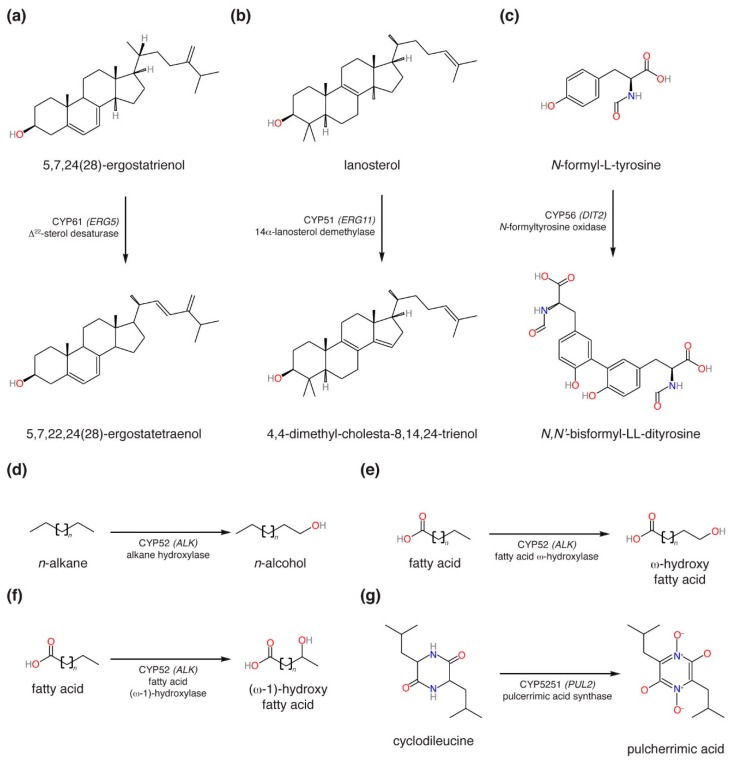
Currently known cytochrome P450 monooxygenase (CYP) enzymatic activities among budding yeasts: (**a**) Δ^22^-sterol desaturase; (**b**) 14α-lanosterol demethylase; (**c**) *N*-formyl-l-tyrosine oxidase; (**d**) *n*-alkane hydroxylase; (**e**) fatty acid ω-hydroxylase; (**f**) fatty acid (ω-1)-hydroxylase; (**g**) pulcherrimic acid synthase.

Among the budding yeasts there are currently five CYP families whose functions have been established through biochemical or genetic means, or both. The CYP51 and CYP61 family enzymes lanosterol 14α-demethylase and Δ^22^-sterol desaturase (encoded by the genes *ERG11* and *ERG5*, respectively) are components of the ergosterol biosynthetic pathway [9,10] (Figure 1a,b). The *DIT2* gene encodes a CYP56 family enzyme involved in the synthesis of ll-*N*,*N’*-bisformyl dityrosine [9] (Figure 1c), which is a component of the outer spore wall [10]. The CYP52 family consists of alkane and fatty acid hydroxylases (Figure 1d–f, encoded by the *ALK* genes), which are involved in the assimilation of alkanes as carbon sources [11] as well as synthesis of extracellular biosurfactants called sophorolipids [12]. Finally, the CYP5251 family consists of pulcherrimic acid synthase (Figure 1g, encoded by the *PUL2* gene), which is involved in the biosynthesis of the siderophore pulcherrimin [13,14]. In addition to these five CYP families, yeast “orphan” CYP families have been identified whose functions remain to be determined. Previously reported orphan CYP families among budding yeasts include CYP501, CYP504, CYP548, CYP5217, CYP5223 and CYP5252 [15,16,17].

The aim of the present study was to conduct a comprehensive survey of CYP genes among budding yeast genomes to firstly identify what CYP families occur within the sub-phylum *Saccharomycotina* and secondly establish how these families are distributed between different budding yeast lineages. This information will provide an overview that will ultimately guide future efforts to establish functions of budding yeast orphan CYPs.

## 2. Materials and Methods

### 2.1. Sequence Retrieval

A core set of budding yeast cytochrome P450 monooxygenase (CYP) protein sequences in GenBank were collected through a PSI-BLAST search using the *Sac. cerevisiae* Erg5 sequence (GenBank protein accession NP_013728) as a query against the nr and refseq_protein databases constrained to sub-phylum *Saccharomycotina* (taxid:147537) with the *E* value inclusion threshold set to 10^−10^. The search was repeated until no new sequences were identified. Identification of CYP protein sequences in the species *Blastobotrys adeninivorans*, *Lachancea kluyveri*, *Lachancea waltii*, *Nakaseomyces bacillisporus*, *Nakaseomyces bracarensis*, *Nakaseomyces castellii*, *Nakaseomyces delphensis*, *Nakaseomyces nivariensis*, and *Torulaspora microellipsoides* were carried out using the GRYC BLAST server (http://igenolevures.org/). CYP families that appeared to be absent in annotated yeast genomes through BLASTP searches were further confirmed by TBLASTN searches against the GenBank reference sequence genomic database and the whole genome shotgun contig (wgs) database (*E* value cutoff 10^−6^) using representative query sequences for each CYP family (Table 1). In yeast species whose genomes currently lack gene annotations, CYP protein sequences were identified through TBLASTN searches against the GenBank wgs database (*E* value cutoff 10^−6^) using representative query sequences for each CYP family (Table 1). Protein sequences were extracted from genomic data using the NCBI ORFfinder server (https://www.ncbi.nlm.nih.gov/orffinder/) with the corresponding translation table selected. All protein sequences were checked for possible prediction errors such as N-terminal truncations or extensions as well as intron prediction artifacts by multiple sequence alignment with MAFFT [18] (http://mafft.cbrc.jp/alignment/server/index.html) using the G-INS-i alignment strategy. CYP sequences that displayed sequence identity equal or above 40% when aligned to the CYP query sequence were automatically assigned to the same CYP family as the query sequence. CYP sequences that displayed sequence identity below 40% when aligned to the CYP query sequence were subjected to phylogenetic analysis to ascertain its CYP genealogy as described in the section below.

### 2.2. Phylogenetic Analysis

Protein sequences were aligned in MAFFT as described above. Selection of sequence positions suitable for phylogenetic analysis was carried out in GBlocks [19] (http://molevol.ibmb.csic.es/Gblocks_server/) with the settings for smaller final blocks and less strict flanking positions enabled. The resulting amino acid positions were then used to construct maximum likelihood trees using PhyML v. 3 [20] (http://www.atgc-montpellier.fr/phyml/) with 1000 bootstrap replicates. The substitution model for each phylogenetic analysis was estimated using SMS [21]. All phylogenetic analyses within the present study converged on a LG substitution model [22] with a proportion of invariant sites and gamma-distributed substitution rates (I + Γ). Trees were visualized using FigTree v. 1.4.2 (http://tree.bio.ed.ac.uk/software/figtree/).

## 3. Results

### 3.1. Sequence Collection and Initial Sorting

**Figure 2:**
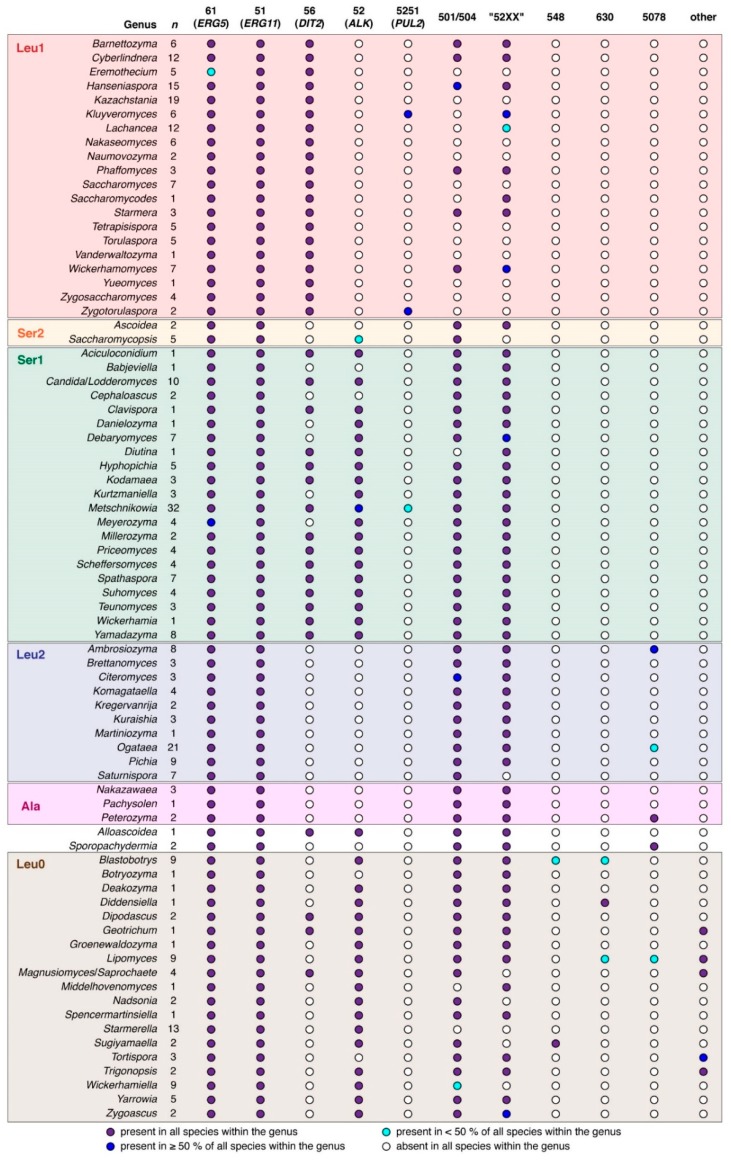
Taxonomic distribution of CYP families among the budding yeasts. Genera are organized with respect to clade [24]. The number of species surveyed within each genus (*n*) is indicated. The color of circles represent the occurrence of each CYP category (either individual CYP families or larger clusters of related CYP families) within each individual genus. Suspected pseudogenes were scored as “absent”. The CYP category “other” denotes CYP families detected exclusively in a small number of basal budding yeast genera. These CYP families are described in greater detail in Table 2.

Compilation of budding yeast CYP protein sequences were carried out in two phases. The first phase involved the collection of budding CYP sequences within the NCBI GenBank protein database using a PSI-BLAST search with the *Sac. cerevisiae* Erg5 sequence as the query. This core set of budding yeast CYP sequences were then preliminarily sorted into CYP families based on a 40% sequence identity threshold against the CYP reference sequences in Table 1. Any protein sequences that did not satisfy the 40% sequence identity threshold were further queried against the *Aspergillus nidulans* protein complement within GenBank to identify matches against the *Asp. nidulans* CYPome reference set [23]. The second phase consisted of TBLATSN searches using the CYP reference proteins against individual budding yeast genomes. Significant hits were translated in silico and sorted as described above. In order to place the budding yeast CYP family inventory in a meaningful taxonomic context, a higher-order classification scheme based on the translation of the codon CUG [24], which is roughly equivalent to the taxonomic rank of order, was used. The budding yeast CYP gene inventory (CYPome) at the taxonomic rank of genus is summarized in Figure 2.

Sequences belonging to families CYP51 (*ERG11*), CYP52 (*ALK*), CYP56 (*DIT2*), CYP61 (*ERG5*) and CYP5251 (*PUL2*) could be assigned with high confidence. The CYP51 family, which is represented by the essential ergosterol biosynthetic enzyme lanosterol 14α-demethylase [25], was detected in all surveyed genomes. CYP61, which is represented by the non-essential ergosterol biosynthetic enzyme Δ^22^-sterol destaurase [26], was detected in all but the three species *Eremothecium coryli*, *Eremothecium cymbalariae* and *Eremothecium sinecaudum*. Both the CYP51 and CYP61 families would typically be represented by a single gene copy per haploid genome. Some notable exceptions included *Alloascoidea hylecoeti* (three CYP51/*ERG11* gene copies), *Blastobotrys proliferans* (at least six CYP61/*ERG5* gene copies) and *Ogataea trehaloabstinens* (three CYP61/*ERG5* and two CYP51/*ERG11* gene copies, respectively).

The CYP56 family (*DIT2*) was ubiquitous in the Leu1 clade, occurred in the majority of genera within the Ser1 clade but was completely absent in clades Ala, Leu2 and Ser2. There were sporadic occurrences of CYP56/*DIT2* in the basal Leu0 clade. In all *DIT2*-containing genomes, the *DIT2* gene co-occurred together with *DIT1* gene in an antiparallel orientation so that the two genes shared a common promoter. The *Geotrichum candidum* genome contained a second *DIT1* copy (GenBank protein accession CDO53799), which did not co-occur with a *DIT2* gene. All yeast genomes that lacked the *DIT2* gene also lacked the *DIT1* gene.

**Figure 3:**
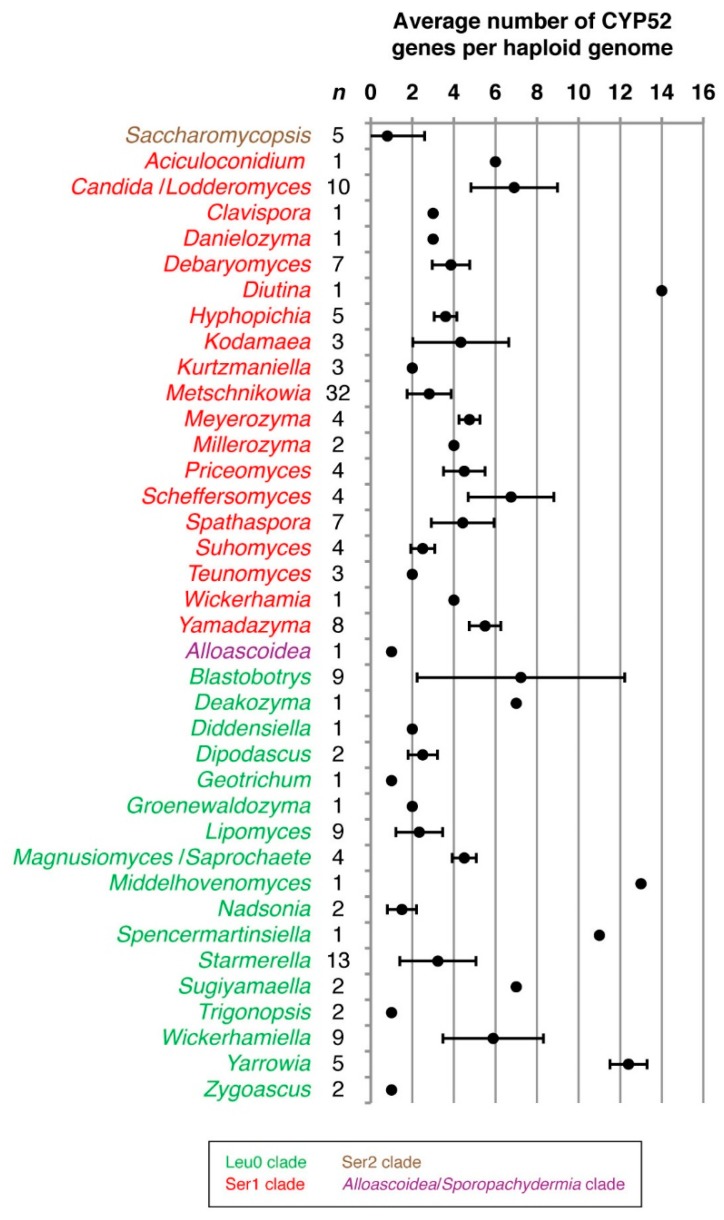
Average gene copy number of CYP52 family genes per haploid genome among budding yeasts. Genus names are color-coded with respect to clade [24]. The number of species surveyed within each genus (*n*) is indicated. Error bars indicate one standard deviation.

The CYP52 family (*ALK*) occurred with high frequency in clades Leu0 and Ser1 while being completely absent in clades Ala, Leu1 and Leu2. Interestingly, only one of seven species within the Ser2 clade (encompassing families Ascoideaceae and Saccharomycopsidaceae)—*Saccharomycopsis fodiens*—possessed CYP52-encoding genes. In terms of gene copy number, the CYP52 family displayed the highest gene copy numbers of all budding yeast CYP families with over ten gene copies per haploid genome observed in several species (Figure 3).

Family CYP5251 (*PUL2*) displayed only sporadic occurrences among budding yeast genomes, which is consistent with previous reports [13]. As expected, the *PUL2* gene co-occurred with the *PUL1* gene, which is thought to catalyze the first step of pulcherrimin synthesis through the conversion of leucine into cyclodileucine [14].

Orphan families CYP501 and CYP504 appeared closely related and in some cases protein sequences could not be unambiguously assigned to either CYP family based on the 40% sequence identity threshold. Such was the case with the *Geo. candidum GECA02s04597g* gene product (GenBank protein accession CDO52018), which displayed 36% and 28% sequence identity to the CYP504 and CYP501 reference sequences, respectively. Some more divergent sequences were even more difficult to assign as in the case of the predicted products of the *Wickerhamiella sorbophila B9G98_03152* and *Pachysolen tannophilus PACTADRAFT_3984* genes (GenBank protein accessions XP_024665477 and ODV94023, respectively), which displayed essentially equal sequence identities to either CYP reference sequence for families CYP501 and CYP504. Therefore, all sequences displaying affinity to either the CYP501 or CYP504 families were sorted into a single category for further characterization by phylogenetic analysis.

A similar dilemma arose when attempting to assign CYP sequences to orphan families CYP5217, CYP5223 and CYP5252. For example, the *Lipomyces starkeyi LIPSTDRAFT_70552* gene product (GenBank protein accession ODQ73543) displayed roughly equal sequence identity (32–34%) to the CYP reference sequences for families CYP5217, CYP5223 and CYP5252. The close relationship between these three CYP families therefore necessitated a common sorting bin, which was designated “CYP52XX” to accommodate all three CYP families as well as any closely related sequences that could not be unambiguously assigned to any of the three families. (It should be noted that the founding member of family CYP5252, which is found in the genome of *Kluyveromyces lactis*—GenBank protein accession XP_453580—appears to be a pseudogene that has resulted in a predicted protein sequence with a 163-amino acid N-terminal truncation.)

One CYP protein sequence (GenBank protein accession ODV87035) from *Ogataea arabinofermentans* could not be unambiguously assigned to any of the CYP families that had previously been recognized among budding yeasts [15,16,17]. The *O. arabinofermentans* protein sequence was 56% identical and 71% similar to the *Asp. nidulans* hypothetical protein An7359 (GenBank protein accession XP_680628), which has been assigned to the CYP5078 family [23]. TBLASTN searches with the *O. arabinofermentans* protein sequence against translated budding yeast draft genomes revealed putative CYP5078-encoding genes within several species of the genera *Ambrosiozyma*, *Ogataea*, *Peterozyma* and *Sporopachydermia* as well as the single species *Lipomyces suomiensis* within the genus *Lipomyces*.

TBLASTN searches against translated budding yeast draft genomes using representative protein sequences for families CYP548, CYP5217, CYP5223 and CYP5252 consistently identified related but divergent putative CYP sequences in the species *Blastobotrys americana*, *Blastobotrys peoriensis*, *Diddensiella caesifluorescens* and *Lipomyces kononenkoae*. The top *Asp. nidulans* BLASTP hit for all four predicted sequences was the An5837 protein (GenBank protein accession XP_663441) with sequence identities ranging from 31 to 38%. The An5837 protein has previously been assigned to the CYP630 family [23]. Even though the sequence similarity threshold was below 40%, these four sequences were designated “CYP630-like” as the author was reluctant to define any new CYP families in the present study as will be expanded on in the discussion.

The remaining CYP sequences, which could not be directly assigned to any of the previously described CYP families in the budding yeasts (and therefore collectively referred to as “other” in Figure 2), were all found in species from basal lineages in the Leu0 clade such as *Geo. candidum*, *Lip. starkeyi* and *Tortispora caseinolytica*. These CYP sequences could be resolved into at least six groups based on simple pairwise similarity comparisons (Table 2). TBLASTN searches against budding yeast draft genomes in the NCBI wgs database confirmed that these sequences appeared to be exclusive to the basal clade Leu0 families Dipodascaceae, Lipomycetaceae and Trigonopsidaceae with high-scoring TBLASTN hits against draft genomes the genera *Lipomyces*, *Magnusiomyces*/*Saprochaete* and *Trigonopsis* Three of the groups (II, IV and VI in Table 2) could be directly assigned to CYP families based on sequence identity to *Asp. nidulans* CYPs [23]. Two additional groups (I and III) could be associated with previously described CYPs in *Asp. nidulans* while the last group (V) was too divergent to be able to confidently assign a tentative CYP family association. Of the six groups identified, group VI (family CYP505) was particularly worthy of mention as this family comprises class III CYPs where an N-terminal fatty acid hydroxylase domain is directly fused to a C-terminal CPR domain [27], which makes the enzyme catalytically self-sufficient [28]. In the filamentous fungus *Fusarium oxysporum*, the CYP505 gene product (P450foxy, GenBank protein accession BAA82526) catalyzes ω-1/ω-3 hydroxylation of fatty acids [28]. The only other budding yeast genomes besides *Lip. starkeyi* to possess a CYP505-encoding gene were those of the closely related species *Lip. kononenkoae* and *Lipomyces lipofer*. It should be noted that the production of extracellular ω-3 hydroxylated fatty acids—so-called oxylipins—have previously been reported in species belonging to the family Lipomycetaceae [29,30].

### 3.2. Phylogenetic Analyses

With the exception of families CYP51 and CYP61, all remaining CYP families and associated sequences were not ubiquitous throughout the budding yeast sub-phylum. Phylogenetic analysis was therefore employed to investigate whether individual CYP families appeared to have been lost in specific lineages or whether there was any evidence that they had been acquired through horizontal gene transfer (HGT) events. In addition, the current study found that the 40% protein sequence identity threshold for CYP family assignment had not been able to confidently classify a significant number of predicted CYP protein sequences. Phylogenetic analysis was therefore used in this context to better resolve predicted CYP protein sequences into previously described CYP families.

**Figure 4:**
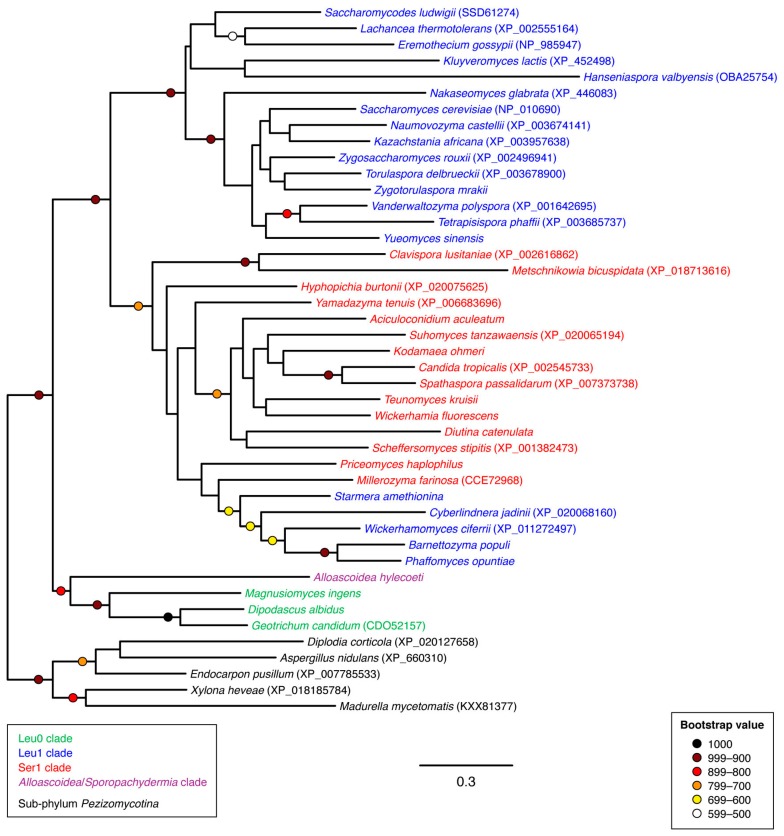
Unrooted phylogram of CYP56 gene products in representative species of sub-phylum *Saccharomycotina* as well as selected non-*Saccharomycotina* species. Individual species are color-coded with respect to clade [24]. The analysis was based on 327 aligned amino acid positions of the corresponding proteins. Colored circles represent branch support with the color indicating the proportion of retained nodes among 1000 bootstrap replicates. Branches lacking circles indicate branch support less than 500. Accession numbers for individual protein sequences are displayed when available. Protein sequences lacking accession numbers were derived through conceptual translation of genomic sequences. (Genomic coordinates for protein sequences derived through conceptual translation of genomic sequences are listed in Table A1 of Appendix A).

Phylogenetic analysis of CYP56 family protein sequences (Figure 4) was consistent with the presence of a CYP56 family gene (*DIT2*) in the common ancestor of budding yeasts with subsequent loss of the *DIT2* gene from several lineages such as the entirety of clades Ala, Leu2 and Ser2 as well as individual genera within the Leu0 clade. The function of the Dit2 *N*-formyl l-tyrosine oxidase in *Sac. cerevisiae* is to synthesize a dityrosine precursor that then becomes incorporated into the outer spore wall [9,10]. The *DIT2* gene has also been shown to be essential for chlamydospore formation in *Candida albicans* [31]. The absence of *DIT2* homologs in the genomes of genera within clades Ala, Leu0, Leu2 and Ser2 would therefore predict that the mature spore from these lineages lack an outer dityrosine layer. However, the author was unable to find any literature regarding spore wall composition in any CYP56-negative lineages.

**Figure 5:**
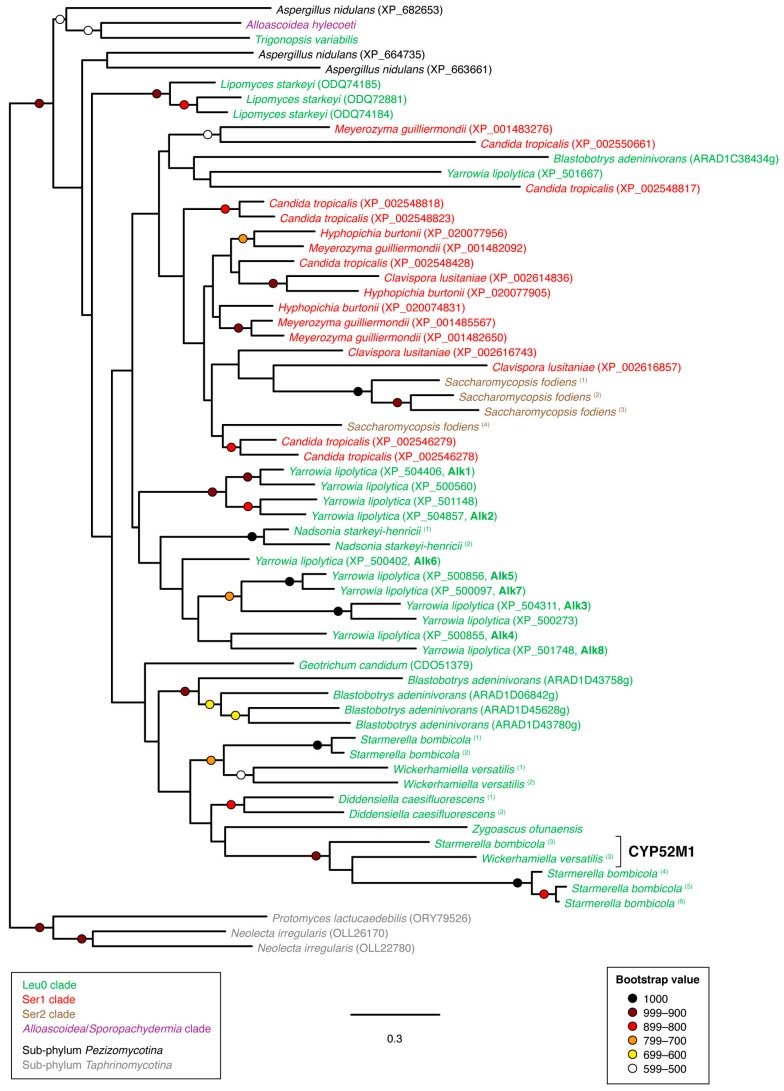
Unrooted phylogram of CYP52 gene products in representative species of sub-phylum *Saccharomycotina* as well as selected non-*Saccharomycotina* species. Individual species are color-coded with respect to clade [24]. The analysis was based on 309 aligned amino acid positions of the corresponding proteins. Colored circles represent branch support with the color indicating the proportion of retained nodes among 1000 bootstrap replicates. Branches lacking circles indicate branch support less than 500. Accession numbers for individual protein sequences are displayed when available. Protein sequences lacking accession numbers were derived through conceptual translation of genomic sequences. Named protein sequences are indicated in bold font. (Genomic coordinates for protein sequences derived through conceptual translation of genomic sequences are listed in Table A1 of Appendix A).

Budding yeast protein sequences belonging to the CYP52 family could not be resolved into a single, well-supported clade by phylogenetic analysis (Figure 5). The relatively high rate of evolution in this particular CYP family made resolution of relationships difficult overall as reflected by the low number of strongly supported tree nodes.

**Figure 6:**
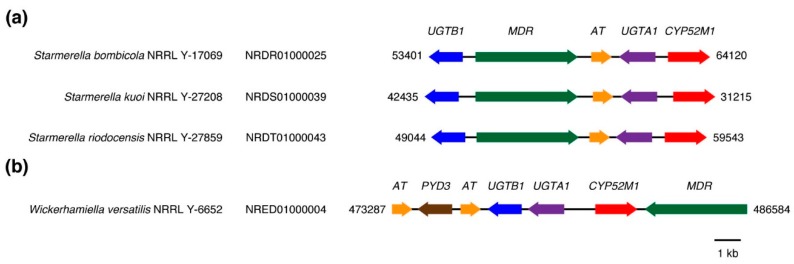
CYP-containing sophorolipid biosynthetic gene clusters in *Starmerella* and *Wickerhamiella* species. Genomic accession numbers and corresponding genomic sequence coordinates are indicated. (**a**) conserved sophorolipid biosynthetic gene clusters within the genus *Starmerella*; (**b**) putative sophorolipid biosynthetic gene cluster in the species *W. versatilis*.

Sophorolipid synthesis in the yeast *Starmerella bombicola* has been shown to involve a specific CYP52 gene, which has been designated CYP52M1 (GenBank protein accession ACD75398) [12]. It has further been shown that the *Sta. bombicola* CYP52M1-encoding gene is situated within a sophorolipid biosynthetic gene cluster [32], which also encodes a putative sophorolipid efflux pump (GenBank protein accession AET14838), an acetyltransferase (GenBank protein accession AEK28753) [33] and two UDP-glucosyltransferases that are the products of the genes *UGTA1* (GenBank protein accession ADT71702) [34] and *UGTB1* (GenBank protein accession ADT71703) [35]. Homologous sequences to *UGTA1* and *UGTB1* could be detected in two other species of *Starmerella* as well as in *Wickerhamiella versatilis*. In all three species, the UDP-glucosyltransferase homologs were also located in putative sophorolipid biosynthetic gene clusters (Figure 6a,b). In the case of the gene cluster in *W. versatilis*, there appeared to have been some rearrangement of the component genes, which included a second acetyltransferease gene (Figure 6b). The putative sophorolipid biosynthetic gene cluster in *W. versatilis* also contained a putative β-ureidopropionase/β-alanine synthase (*PYD3*), which is normally involved in the catabolism of dihydropyrimidines [36]. It remains to be determined whether this gene can also play a role in sophorolipid biosynthesis. Notably, the predicted protein sequence of the *W. versatilis* CYP52-encoding gene within the putative gene cluster formed a well-supported clade that included the *Sta. bombicola* CYP52M1 sequence (Figure 5). It was also notable that no UDP-glucosyltransferase homologs could be detected in the genome of *Starmerella apicola* strain NRRL Y-2481, even though this particular strain has previously been shown to be a significant sophorolipid-producer [37]. One possibility is that *Sta. apicola* employs a different family of glucosyltransferases for sophorolipid biosynthesis. This appears to be the case for the sophorolipid-producing yeast *Wickerhamiella domercqiae*, whose genome also lacks homologs of *UGTA1* and *UGTB1* but has been shown to produce glucose-containing sophorolipids [38]. None of the CYP52-encoding genes in *Sta. apicola* (four copies) or *W. domercqiae* (two copies) were immediately adjacent to a predicted glucosyltransferase, a predicted acetyltransferase or a predicted efflux pump. This would suggest either that sophorolipid biosynthetic genes are not clustered in these two species or that the CYP52 component is not situated within a gene cluster, should they exist.

**Figure 7:**
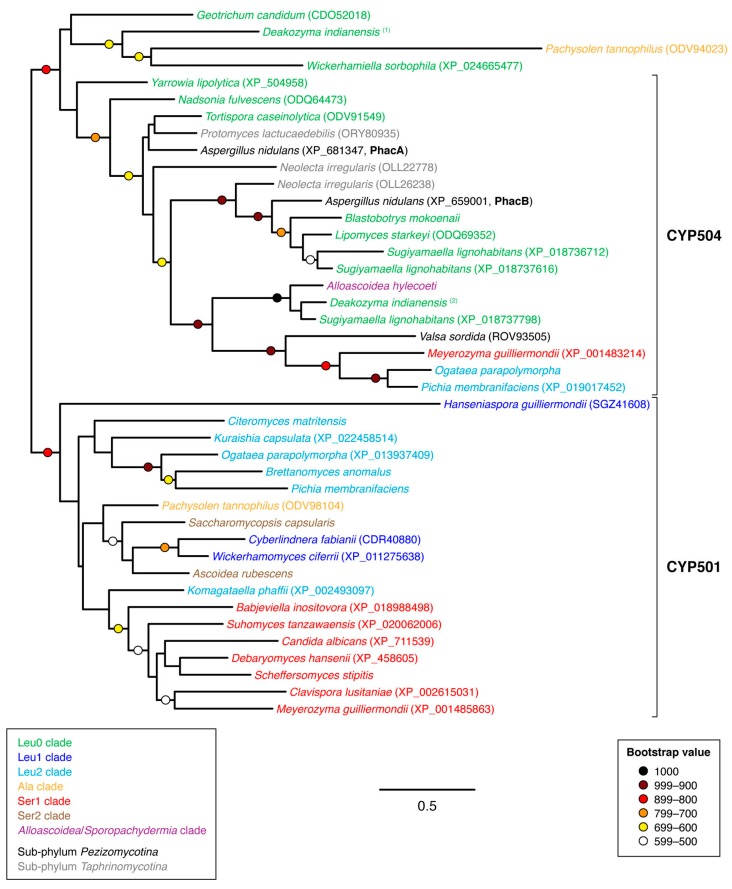
Unrooted phylogram of CYP501 and CYP504 gene products in representative species of sub-phylum *Saccharomycotina* as well as selected non-*Saccharomycotina* species. Individual species are color-coded with respect to clade [24]. The analysis was based on 252 aligned amino acid positions of the corresponding proteins. Colored circles represent branch support with the color indicating the proportion of retained nodes among 1000 bootstrap replicates. Branches lacking circles indicate branch support less than 500. Accession numbers for individual protein sequences are displayed when available. Protein sequences lacking accession numbers were derived through conceptual translation of genomic sequences. Named protein sequences are indicated in bold font. (Genomic coordinates for protein sequences derived through conceptual translation of genomic sequences are listed in Table A1 of Appendix A).

The families CYP501 and CYP504 could be resolved reasonably well by phylogenetic analysis although some divergent sequences from *Deakozyma indianensis*, *Geo. candidum*, *Pac. tannophilus* and *Wickerhamiella sorbophila* made precise circumscription of each family difficult (Figure 7). Family assignments as shown in Figure 7 were therefore guided by previous assignments of CYP family within the budding yeasts [15,16,17]. At present, there are no published reports on the biological functions of either CYP family in budding yeasts. It was notable that the budding yeast CYP504 protein sequences did not form a monophyletic clade within the family but instead was interspersed with CYP504 protein sequences from the other two ascomycete sub-phyla *Pezizomycotina* and *Taphrinomycotina*. This raises the possibility of potential HGT events between other ascomycete lineages and the budding yeasts.

The *Asp. nidulans* CYP504 genes *phacA* and *phacB* have previously been shown to encode a phenylacetate 2-hydroxylase and a 3-hydroxyphenylacetate/3,4-dihydroxyphenylacetate 6-hydroxylase, respectively [39,40]. Both these enzymes participate in the catabolism of the aromatic amino acids phenylalanine and tyrosine through the homogentisate pathway. These enzymes also enable the use of externally provided phenylacetate and its hydroxylated variants as carbon sources. Catabolism of the carbon skeleton of phenylalanine and tyrosine has yet to be investigated in budding yeasts. Nevertheless the homogentisate pathway enzyme 4-hydroxyphenylpyruvate dioxygenase (HPPD) appears to be conserved in most budding yeast species with the exception of the family Saccharomycetaceae. This could indicate that these two CYP families may play a role in phenylalanine and tyrosine catabolism. However, it should be noted that families CYP501 and CYP504 were absent in the species *Saccharomycodes lugwigii* and *Middelhovenomyces tepae* as well as all surveyed species of the genus *Starmerella* and some species of the genus *Wickerhamiella*, all of which possess a putative HPPD gene. The utilization of phenylacetate and its hydroxylated variants as carbon sources have previously been reported in the genera *Blastobotrys* and *Geotrichum* [41].

**Figure 8:**
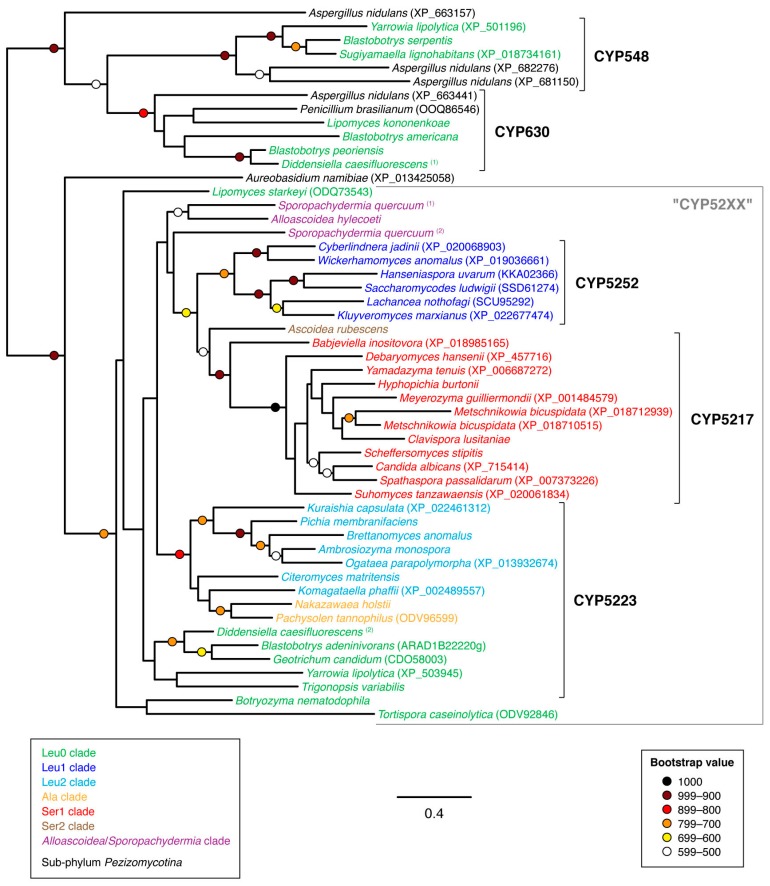
Unrooted phylogram of CYP548, CYP630, CYP5217, CYP5223, CYP5252 and associated gene products in representative species of sub-phylum *Saccharomycotina* as well as selected non-*Saccharomycotina* species. Individual species are color-coded with respect to clade [24]. The analysis was based on 217 aligned amino acid positions of the corresponding proteins. Colored circles represent branch support with the color indicating the proportion of retained nodes among 1000 bootstrap replicates. Branches lacking circles indicate branch support less than 500. Accession numbers for individual protein sequences are displayed when available. Protein sequences lacking accession numbers were derived through conceptual translation of genomic sequences. (Genomic coordinates for protein sequences derived through conceptual translation of genomic sequences are listed in Table A1 of Appendix A).

Families CYP548 and CYP630 displayed moderate sequence similarity with the “CYP52XX” cluster (families CYP5217, CYP5223, CYP5252 and associated sequences) and together could produce a multiple sequence alignment of reasonable quality, which suggests that these families may be neighboring taxa. Family CYP5078 also displayed moderate sequence similarity with families CYP548 and CYP630 as well as the “CYP52XX” cluster but could not produce a multiple sequence alignment of sufficient quality to generate an informative phylogeny. The non-overlapping taxonomic distribution of the CYP families CYP5217 (Ser1 clade), CYP5223 (Ala, Leu0 and Leu2 clades) and CYP5252 (Leu1 clade) suggested that all three families were descended from a rapidly evolving budding yeast-specific “proto-CYP”. This was supported by phylogenetic analysis, which produced a well-supported clade of all three CYP families with associated sequences from more basal budding yeast taxa (Figure 8). Families CYP548 and CYP630 also displayed well-supported clades adjacent to the “CYP52XX” cluster. Since both CYP548 and CYP630 were exclusive to genera within the basal Leu0 clade of the budding yeasts, this suggested that both were present in the last common ancestor of all budding yeasts followed by subsequent loss of both CYP families within the majority of clades.

**Figure 9:**
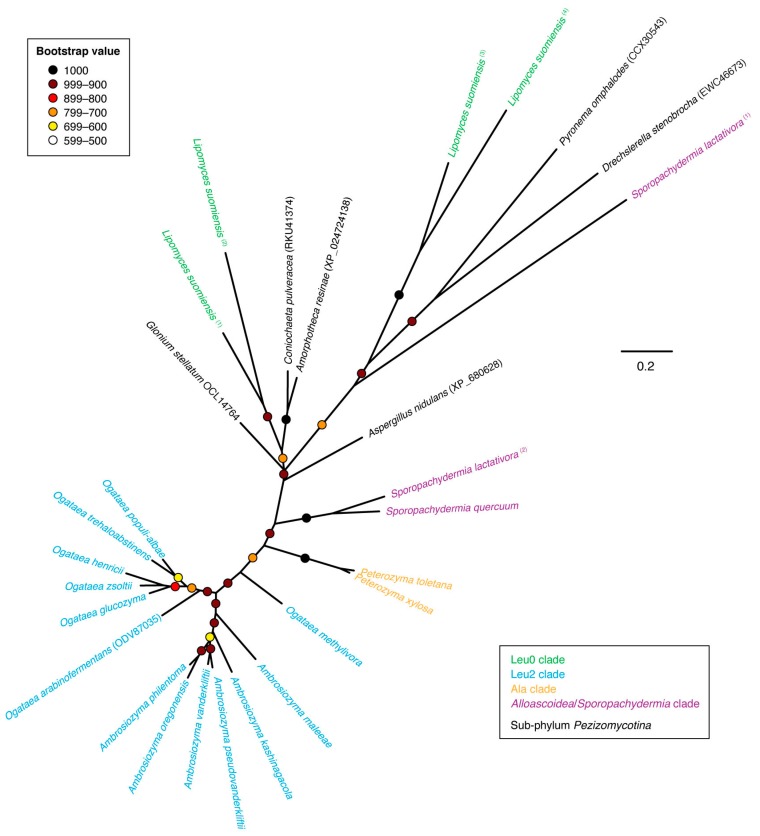
Unrooted tree of CYP5078 gene products in representative species of sub-phylum *Saccharomycotina* as well as selected non-*Saccharomycotina* species. Individual species are color-coded with respect to clade [24]. The analysis was based on 403 aligned amino acid positions of the corresponding proteins. Colored circles represent branch support with the color indicating the proportion of retained nodes among 1000 bootstrap replicates. Branches lacking circles indicate branch support less than 500. Accession numbers for individual protein sequences are displayed when available. Protein sequences lacking accession numbers were derived through conceptual translation of genomic sequences. (Genomic coordinates for protein sequences derived through conceptual translation of genomic sequences are listed in Table A1 of Appendix A).

Phylogenetic analysis of CYP5078 family gene products failed to produce a monophyletic clade encompassing all budding yeasts to the exclusion of other ascomycete fungi (Figure 9). In fact, budding yeast CYP5078 proteins were separated into four well-supported clades interspersed with non-*Saccharomycotina* sequences. This raises the possibility that some of these occurrences within the sub-phylum *Saccharomycotina* may be the result of HGT events from filamentous ascomycetes (sub-phylum *Pezizomycotina*).

## 4. Discussion

The purpose of the current study was to map the budding yeast CYPome in order to gain a better understanding of CYP family diversity and distribution within this sub-phylum. With this information in hand, it will now be possible to design a more targeted strategy for functional characterization of orphan CYPs among budding yeasts. The updated list of orphan CYP families within sub-phylum *Saccharomycotina* (including basal lineages) currently stands at 14 CYP family (or CYP family-like) categories: CYP59-like, CYP501, CYP504, CYP505 (class III putative fatty acid ω-1/ω-3 hydroxylase), CYP540, CYP548, CYP617-like, CYP630, CYP677, CYP5078, CYP5217, CYP5223 and CYP5252 as well as the divergent “group V” CYPs in basal budding yeast lineages (Table 2). Based on phylogenetic analysis, families CYP5217, CYP5223 and CYP5252 form a well-supported monophyletic clade, suggesting accelerated evolution within these CYP families. Phylogenetic analysis also revealed some notable instances where budding yeast CYP gene products were not resolved into well-supported monophyletic clades, which was observed in families CYP52 (Figure 5), CYP504 (Figure 7) and CYP5078 (Figure 9). The current dataset was not robust enough to make a confident determination on whether the observed topologies are the result of selective gene losses or HGT events. Further genomic sequences will hopefully shed more light on these observations.

The exceptionally wide substrate range of CYP superfamily enzymes is reflected in the very high degree of sequence variability between individual CYP families. The only significant sequence conservation observed among all CYPs is a set of four motifs dedicated to binding and stabilizing the heme moiety [17]. As a consequence, there is a rapid loss of phylogenetic signal between CYP families, which complicates any effort at reconstructing the evolutionary history between more divergent CYP families. This sequence variability also necessitates highly sensitive sequence analysis tools such as the position-specific scoring matrices employed by the PSI-BLAST algorithm for the identification of novel CYP families. The author does not exclude the possibility that additional CYP families remain to be identified among already sequenced genomes. The sequence retrieval strategy employed in the current study relies extensively on already existing protein sequences within GenBank. Highly divergent CYP families within yeast draft genomes that currently lack gene annotations are therefore likely to be missed using the sequence retrieval approach described here. This is especially the case for basal yeast lineages within the Leu0 clade whose genes are more likely to contain introns and therefore TBLASTN searches against such genomes are expected to produce truncated hits with lower statistical significance than would be expected from sequence similarity searches against genomes containing predominantly single-exon genes. It is therefore essential that all budding yeast genomes be properly annotated and that the predicted gene products of each genome be included in the GenBank protein database.

Throughout the present study, the author has been reluctant to define and name new CYP families as it may be counterproductive to functional annotation efforts. The designation of CYP family carries the implication that members within the same family broadly share the same enzymatic function while those sequences below the 40% sequence identity threshold do not. At present, there is no experimental data to either support or reject this notion. An additional classification system has been described that further classifies CYP families into clans [42] but the criteria for inclusion within a specific clan are less well defined. The utility of the current CYP nomenclature system for the assignment of gene function is also severely limited by the general lack of genetic and biochemical data for the vast majority of CYP families.

As demonstrated by the apparent monophyly of families CYP5217, CYP5223 and CYP5252 within the budding yeasts (Figure 8), the enthusiasm to name new CYP families in the absence of phylogenetic analysis can give a misleading impression of CYP family diversity, especially in the case of rapidly evolving CYP families. The CYP family naming convention also becomes problematic in the case of yeast lineages that display accelerated rates of evolution. For example, a sub-set of species within the genus *Hanseniaspora* has been reported to evolve at a faster rate following losses of genes involved with genome integrity [43]. The CYP501-like gene product (GenBank protein accession SGZ41608) from one such species—*Hanseniaspora guilliermondii*—displayed only 25% sequence identity with CYP501 reference sequence from *Meyerozyma guilliermondii*, yet the *Han. guilliermondii* sequence could be confidently placed within the CYP501 clade through phylogenetic circumscription (Figure 7). Likewise, the CYP5252-like gene product from *Hanseniaspora uvarum* (GenBank protein accession KKA02366) displayed 36% sequence identity with the *Cyberlindnera fabianii* CYP5252 reference sequence but phylogenetic analysis placed the *Han. uvarum* sequence confidently within the CYP5252 family clade (Figure 8).

The results presented in the current study would argue for a CYP family classification system based on phylogenetic circumscription similar to what has previously been proposed for the clan nomenclature system. The author does not suggest a full-scale revamp of the CYP nomenclature system but would nevertheless stress the prioritization of functional characterization of already named CYP families over the continued rush to name new CYP families among the exponentially growing number of sequenced genomes. Functional characterization by biochemical and genetic means will ultimately enable better evaluation of the current CYP nomenclature system.

From a biotechnological view, budding yeasts may seem like a poor source of novel CYPs for industrial and synthetic biology applications. However, it precisely the scarcity of endogenous CYPs that have made budding yeasts a good heterologous expression system for functional characterization of eukaryotic CYPs [44]. The common baker’s yeast *Sac. cerevisiae* is by far the most versatile eukaryotic model system today and contains only three endogenous CYPs—CYP61 (*ERG5*), CYP51 (*ERG11*) and CYP56 (*DIT2*). This fact makes *Sac. cerevisiae* a good synthetic biology platform for production of bioactive compounds whose biosynthesis depend CYP activity as there is little risk of unwanted side reactions caused by endogenous CYP activity.

## Figures and Tables

**Table 1:** CYP family reference sequences.

CYP Family	Species	Accession Number
51 (*ERG11*)	*Saccharomyces cerevisiae*	NP_011871
52 (*ALK*)	*Candida tropicalis*	XP_002546278
56 (*DIT2*)	*Saccharomyces cerevisiae*	NP_010690
61 (*ERG5*)	*Saccharomyces cerevisiae*	NP_013728
501	*Meyerozyma guillermondii*	XP_001485863
504	*Meyerozyma guillermondii*	XP_001483214
548	*Yarrowia lipolytica*	XP_501196
5217	*Candida albicans*	XP_715414
5223	*Yarrowia lipolytica*	XP_503945
5251 (*PUL2*)	*Kluyveromyces lactis*	XP_453057
5252	*Cyberlindnera fabianii*	CDR39009

**Table 2:** CYP genes in basal budding yeast taxa.

Species	Protein Accession	Top Assigned *Asp. nidulans* CYP Hit (Identity/Similarity)
**Group I (CYP617-like)**		
*Lipomyces starkeyi*	ODQ70065	XP_659488 (31%/50%)
*Lipomyces starkeyi*	ODQ69649	XP_659488 (32%/50%)
*Lipomyces starkeyi*	ODQ71312	XP_659488 (32%/51%)
*Lipomyces starkeyi*	ODQ70063	XP_659488 (34%/55%)
*Lipomyces starkeyi*	ODQ72725	XP_659488 (34%/51%)
*Tortispora caseinolytica*	ODV92859	XP_659488 (27%/47%)
**Group II (CYP540)**		
*Lipomyces starkeyi*	ODQ75312	XP_682188 (46%/65%) ^1^
**Group III (CYP59-like)**		
*Lipomyces starkeyi*	ODQ74007 ^2^	XP_681077 (38%/55%) ^3^
**Group IV (CYP677)**		
*Lipomyces starkeyi*	ODQ75272	XP_681884 (44%/60%)
**Group V**		
*Geotrichum candidum*	CDO53625	XP_661721 (22%/41%)
*Lipomyces starkeyi*	ODQ69471	XP_661721 (23%/40%)
*Lipomyces starkeyi*	ODQ74469	XP_661721 (23%/36%)
*Lipomyces starkeyi*	ODQ69396	XP_660953 (27%/45%)
*Lipomyces starkeyi*	ODQ72272	XP_661721 (22%/40%)
*Lipomyces starkeyi*	ODQ72285	XP_682522 (31%/48%)
**Group VI (CYP505)**		
*Lipomyces starkeyi*	ODQ75335	XP_664439 (46%/58%)

^1^ The top *Asp. nidulans* hit overall (43%/59%) is a hypothetical CYP (XP_663370), which has not been assigned a CYP family [23] and the corresponding gene (*AN3861*) is not currently listed in the *Aspergillus* genome database (http://www.aspergillusgenome.org/). ^2^ Potentially truncated N-terminus due to suspected gene prediction error. ^3^ The top *Asp. nidulans* hit overall (56%/73%) is a hypothetical CYP (XP_661465), which has not been assigned a CYP family [23] and the corresponding gene (*AN5766*) is not currently listed in the *Aspergillus* genome database.

## Appendix A

**Table A1:** Genomic sequences used for conceptual translation into protein sequences for phylogenetic analysis. Numbers in parentheses refers to numbered sequences in the corresponding figures.

Species	Genomic Accession ^1^	Strand	Coordinates
CYP56			
*Aciculoconidium aculeatum*	PPJB01000050 ^2^	+	74966–76441
*Alloascoidea hylecoeti*	BCKZ01000006	−	381233–382128, 382188–382782
*Barnettozyma populi*	PPLZ02000015	+	151344–152804
*Dipodascus albidus*	PPJE01000011	+	4500–5072, 5133–5489, 5549–6016, 6083–6148
*Diutina catenulata*	PJEZ01000006 ^2^	+	67720–69054
*Kodamaea ohmeri*	PPNN01000011 ^2^	−	275828–277339
*Magnusiomyces ingens*	UIDE01000004	−	435148–435696, 435779–436135, 436192–436770
*Priceomyces haplophilus*	BCIF01000002 ^2^	+	1110008–1111489
*Phaffomyces opuntiae*	PPNH01000004	−	4783–6303
*Starmera amethionina*	PPNC01000057	+	57884–59377
*Teunomyces kruisii*	PPLR01000028 ^2^	+	76692–78167
*Wickerhamia fluorescens*	BCGE01000005 ^2^	−	792413–793876
*Yueomyces sinensis*	PPMT01000005	−	193708–195165
*Zygotorulaspora mrakii*	PPHZ01000005	+	324089–325603
CYP52			
*Alloascoidea hylecoeti*	BCKZ01000001	−	888959–890518
*Diddensiella caesifluorescens* (1)	PPJD01000017	+	195224–196783
*Diddensiella caesifluorescens* (2)	PPJD01000017	+	197357–198934
*Nadsonia starkeyi-henricii* (1)	QBLK01000108	−	23137–24687
*Nadsonia starkeyi-henricii* (2)	QBLK01000108	−	21081–22637
*Saccharomycopsis fodiens* (1)	JNFV01000009 ^2^	−	683357–684952
*Saccharomycopsis fodiens* (2)	JNFV01000003 ^2^	+	6643–8226
*Saccharomycopsis fodiens* (3)	JNFV01000009 ^2^	+	7380–8951
*Saccharomycopsis fodiens* (4)	JNFV01000001 ^2^	+	2891–4453
*Starmerella bombicola* (1)	NRDR01000004	−	591579–593135
*Starmerella bombicola* (2)	NRDR01000004	−	318040–319596
*Starmerella bombicola* (3)	NRDR01000025	+	62522–64117
*Starmerella bombicola* (4)	NRDR01000003	−	308477–310045
*Starmerella bombicola* (5)	NRDR01000005	−	9970–11538
*Starmerella bombicola* (6)	NRDR01000005	−	6608–8176
*Trigonopsis variabilis*	PPXM02000002	−	1137995–1139512
*Wickerhamiella versatilis* (1)	NRED01000001	−	1258441–1259970
*Wickerhamiella versatilis* (2)	NRED01000011	−	25516–27039
*Wickerhamiella versatilis* (3)	NRED01000004	+	480766–482298
*Zygoascus ofunaensis*	PPMC02000012	−	276041–277603
CYP501, CYP504			
*Alloascoidea hylecoeti*	BCKZ01000004	−	123913–125610
*Ascoidea rubescens*	NW_017962913 ^2^	+	1016180–1017844
*Blastobotrys mokoenaii*	PPJM02000008	−	17335–18903
*Brettanomyces anomalus*	LCTY01000003	−	955844–957598
*Citeromyces matritensis*	PPHV01003366	+	46403–47908
*Deakozyma indianensis* (1)	PPLG02000002	+	204898–206562
*Deakozyma indianensis* (2)	PPLG02000014	−	73651–75282
*Ogataea parapolymorpha*	NC_027864	−	1260033–1261667
*Pichia membranifaciens*	NW_017566986	−	510782–512500
*Saccharomycopsis capsularis*	PPIG01000060 ^2^	+	55413–56858
*Scheffersomyces stipitis*	NC_009047 ^2^	−	132488–134149
CYP548, CYP630, CYP5217, CYP5223, CYP5252			
*Alloascoidea hylecoeti*	BCKZ01000023	−	227089–228648
*Ambrosiozyma monospora*	BCIP01000010	−	36354–38219
*Ascoidea rubescens*	NW_017962915 ^2^	−	986327–988084
*Blastobotrys americana*	PPJN02000012	+	34235–35698
*Blastobotrys peoriensis*	PPJJ02000009	−	104592–106094
*Blastobotrys serpentis*	PPJG01000004	−	306605–308191
*Botryozyma nematodophila*	PPJC01001041	−	443–1888
*Brettanomyces anomalus*	LCTY01000007	+	286500–288059
*Citeromyces matritensis*	PPHV01003001	−	5839–7305
*Clavispora lusitaniae*	NW_003101576 ^2^	−	792130–793887
*Diddensiella caesifluorescens* (1)	PPJD01000014	+	6451–7920
*Diddensiella caesifluorescens* (2)	PPJD01000011	−	56654–58084
*Hyphopichia burtonii*	NW_017963729 ^2^	+	1954183–1955925
*Lipomyces kononenkoae*	PPJW01000001	+	112103–112111, 112147–112381, 112443–113550, 113613–113838
*Nakazawaea holstii*	PPKU01000003 ^3^	+	174994–176508
*Pichia membranifaciens*	NW_017566985	+	474706–476289
*Scheffersomyces stipitis*	NC_009047 ^2^	+	1074603–1076360
*Sporopachydermia quercuum* (1)	BCGN01000006	−	1022779–1024251
*Sporopachydermia quercuum* (2)	BCGN01000011	+	318748–320439
*Trigonopsis variabilis*	PPXM02000001	−	657214–658683
CYP5078			
*Ambrosiozyma kashinagacola*	BCGA01000001	+	1475803–1477413
*Ambrosiozyma maleeae*	PPLV01000007	+	15809–17437
*Ambrosiozyma oregonensis*	PPKY02000013	+	3245–4852
*Ambrosiozyma philentoma*	PPKZ02000016	−	79288–80895
*Ambrosiozyma pseudovanderkliftii*	PPLW02000009	−	377701–379308
*Ambrosiozyma vanderkliftii*	PPHW01000114	−	32141–33748
*Lipomyces suomiensis* (1)	PPJQ02000001	+	628395–629990
*Lipomyces suomiensis* (2)	PPJQ02000011	−	223225–224796
*Lipomyces suomiensis* (3)	PPJQ02000011	+	248970–249275, 249341–249604, 249662–250591
*Lipomyces suomiensis* (4)	PPJQ02000063	+	4147–4461, 4542–4781, 4846–5757
*Ogataea glucozyma*	PPKO01000004	+	345713–347290
*Ogataea henricii*	PPHT01000003	+	42457–44139
*Ogataea methylivora*	PPKQ01000003	+	112748–114325
*Ogataea populi-albae*	PPIX02000004	+	115779–117362
*Ogataea trehaloabstinens*	PPKJ01000015	+	25059–26642
*Ogataea zsoltii*	PPKH02000011	−	45513–47093
*Peterozyma toletana*	PPKG01000007 ^3^	−	78966–80543
*Peterozyma xylosa*	PPKF01000009 ^3^	−	77489–79066
*Sporopachydermia lactativora* (1)	PPID01000125	−	23315–24868
*Sporopachydermia lactativora* (2)	PPID01000036	+	26664–28286
*Sporopachydermia quercuum*	BCGN01000002	+	250317–251900

^1^ Translation table 1 was used unless otherwise indicated. ^2^ Translation table 12. ^3^ Translation table 27.
